# Autism in Adulthood: Psychiatric Comorbidity in High-Functioning Autistic Adults in an Outpatient Clinical Population

**DOI:** 10.3390/neurosci6040117

**Published:** 2025-11-18

**Authors:** Martina Pelle, Federico Fiori Nastro, Chiara Maimone, Stefano Malara, Vincenzo Di Lazzaro, Giorgio Di Lorenzo, Michele Ribolsi

**Affiliations:** 1 Unit of Neurology, Neurophysiology, Neurobiology and Psychiatry, Department of Medicine, Campus Bio-Medico University, 00128 Rome, Italy; 2 Fondazione Policlinico Universitario Campus Bio-Medico, 00128 Rome, Italy; 3 Department of Systems Medicine, Tor Vergata University of Rome, 00133 Rome, Italy; 4 IRCCS Fondazione Santa Lucia, 00179 Rome, Italy; 5 Department of Life Science, Health and Health Professions, Link Campus University, 00165 Rome, Italy

**Keywords:** Autism spectrum disorder, comorbidities, differential diagnosis, autism in adulthood, trajectories

## Abstract

Background: Autism Spectrum Disorder (ASD) is a complex and heterogeneous neurodevelopmental condition. Diagnosing ASD in adults, especially in milder forms, remains challenging due to camouflaging strategies, adaptive behaviors, and frequent psychiatric comorbidities. Despite increased awareness, there is a critical need to improve recognition and tailored interventions for adults with ASD. This study aims to examine the prevalence of psychiatric comorbidities among individuals diagnosed with ASD. Methods: This retrospective cross-sectional study examined 64 adults diagnosed with ASD (*n* = 29 females, 45.3%; age: range, 18–57 years; mean ± SD, 30.9 ± 8.92), who accessed two university hospital outpatient units in Rome between September 2023 and January 2025. All participants were assessed using the Autism Diagnostic Observation Schedule, Second Edition–Module 4 (ADOS-2). Psychiatric comorbidities were evaluated using clinical assessments and the Mini-International Neuropsychiatric Interview (M.I.N.I.) Plus. Results: All patients received an ASD diagnosis without intellectual disability. Forty-four (68.8%) presented with at least one psychiatric comorbidity, most commonly depressive (25.0%) and anxiety disorders (9.4%). Over half of the participants (57.4%) reported at least mild depressive symptoms, and 42.6% exhibited moderate to severe depressive levels. Conclusions: High rates of psychiatric comorbidities, particularly mood and anxiety disorders, were observed, underscoring the importance of comprehensive, multidisciplinary assessment and individualized interventions. Further research using larger samples and rigorous methodologies is warranted to better characterize the ASD phenotype in adults and guide targeted therapeutic strategies.

## 1. Introduction

Autism Spectrum Disorder (ASD) is a complex neurodevelopmental condition that affects how individuals perceive and interact with the world. Rather than a single, uniform disorder, autism is now understood as a spectrum of diverse neurodevelopmental profiles, characterized by early-onset difficulties in social communication and unusually restricted repetitive behavior and narrow interests [1,2]. The conceptual shift from multiple single disorders to a unique spectrum reflects the growing recognition that autistic traits can manifest with varying degrees of intensity and in distinct combinations across individuals and across the lifespan.

ASD is estimated to affect around 1–2% of the population worldwide, with a prevalence of 100/10,000 [3] making it a prominent focus of clinical, educational, and policy-related efforts. According to the surveillance program ‘The Autism and Developmental Disabilities Monitoring’ (ADDM), founded by the U.S Centers for Disease Control and Prevention (CDC), prevalence moved from 0.6% in 2000 to 1.4% in 2010 to 2.7% in 2020 [4]. ADDM estimated that 1 in 36 children in the USA has an ASD, and it is 3.8 times as prevalent among boys as among girls. Similar data are reported in Italy, with 1 in 77 children aged between 7 and 9 years diagnosed with ASD, and boys being four times more affected than girls [5]. The growing awareness of ASD in recent decades has contributed to earlier diagnoses and improved support [6], highlighting the limitations of traditional categorical models and the need for more inclusive, individualized approaches [7].

Early diagnosis tends to be made more readily in individuals with severe symptoms (e.g., extreme social aloneness, no eye contact, and frequent motor stereotypes) and concurrent developmental difficulties (e.g., cognitive or language delay). ASD in people with more subtle difficulties tends to be recognized later [8].

The identification of ASD in adulthood is increasingly acknowledged as a significant clinical concern.

ASD identification in adulthood was established for the first time in the Diagnostic and Statistical Manual of Mental Disorders, Fifth Edition (DSM-5) [2]. According to DSM-5 definition: (1) diagnostic behavioral descriptions apply to all ages; (2) behavior contributing to a diagnosis can be current or historical; (3) and the criterion of a specific early age of onset is no longer required, being replaced by “*symptoms must be present in the early developmental period (but may not become fully manifest until social demands exceed limited capacities, or may be masked by learned strategies in later life*.” [2].

This shift reflects enhanced public and professional awareness, the progressive broadening of diagnostic criteria, and the conceptualization of autism as a spectrum, which has extended the understanding of ASD beyond its traditional association with childhood onset. The accurate and timely identification of ASD in adulthood and the provision of appropriate support services have become a critical clinical priority [9,10]. Supportive government policies should underpin the development of supportive, inclusive, and autism-friendly social and physical environments. However, making a first diagnosis of ASD in adults can be challenging. First, the absence of caregivers or informants able to provide reliable information on early developmental history frequently limits the assessment of childhood symptoms. Second, individuals may have developed compensatory, masking, or camouflaging strategies over time, which can obscure core autistic traits and hinder clinical recognition. Third, the frequent presence of neurodevelopmental or psychiatric comorbidities may further complicate the diagnostic process by overlapping with or mimicking ASD symptomatology [11,12,13,14].

The diagnostic process includes referral, screening, interviews with informants and patients, and functional assessments. In delineating differential diagnoses, true comorbidities, and overlapping behaviour with other psychiatric diagnoses, particular attention should be paid to anxiety, depression, obsessive–compulsive disorder, psychosis, personality disorders, and other neurodevelopmental disorders. Possible misdiagnosis, especially in women, should be explored [15].

A study of 859 adults referred for ASD assessment found high rates of psychiatric diagnoses (>57%) in both ASD and non-ASD groups, with anxiety disorders being significantly more prevalent in those with ASD [16]. Another study reported that 24.6% of 1211 ASD adults perceived at least one previous psychiatric diagnosis as a misdiagnosis, with personality disorders being the most frequent [17]. In a sample of 161 adults diagnosed with ASD, there was a median 11-year gap between the first mental health evaluation and ASD diagnosis [18]. These findings suggest that adult psychiatric care may not adequately recognize ASD, particularly in patients with substance abuse and psychiatric symptoms [19], highlighting the need for improved clinician training in adult ASD presentation. This study aims to present original data derived from real-world clinical activities of two adult ASD outpatient services based in Rome. The primary objective is to assess the prevalence of psychiatric comorbidities among individuals diagnosed with ASD. The secondary objective is to explore the presence of depressive symptoms and their correlation with autistic traits.

## 2. Materials and Methods

This study has a retrospective cross-sectional design. Sixty-four subjects (29 females, 45.3%; 35 males, 54.7%; age: range, 18–57 years; mean ± SD, 30.9 ± 8.92) have been enrolled. Data were collected from the psychiatry outpatient units of two University Hospitals in Rome, the Campus Bio-Medico University Hospital and the Tor Vergata University Hospital, between September 2023 and January 2025.

The adopted inclusion criteria for participation in the study were: a confirmed diagnosis of ASD, based on standardized clinical assessment, and the provision of written informed consent by the participant.

The adopted exclusion criteria were: age under 18 or over 60 years old; an intelligence quotient equal to or less than 70, established by the Wechsler Adult Intelligence Scale-Revised (WAIS-R) [20]; concurrent presence of relevant neurological comorbidities (e.g., epilepsy, concussion, or traumatic brain injury); and current substance use disorder.

The study was approved by the Independent Ethics Committee of Policlinico Tor Vergata (#184/25; 26 June 2025), and informed consent was obtained from all participants.

All subjects enrolled underwent clinical assessment and diagnostic evaluation, conducted by expert psychiatrists. Sociodemographic data (including age, sex, and marital status) and clinical information were systematically collected.

All subjects enrolled met the DSM-5 criteria for ASD diagnosis (Level 1 ASD) [2]. All participants were assessed using the Autism Diagnostic Observation Schedule, Second Edition–Module 4 (ADOS-2) [21], a standardized semi-structured assessment tool to evaluate social interaction and communication, play, and restricted or repetitive behaviors. The ADOS-2 was administered by expert clinicians familiar with its use. Psychiatric comorbidities were diagnosed through clinical evaluation and the Mini-International Neuropsychiatric Interview (M.I.N.I.) Plus [22], which assesses 26 psychiatric disorders following the classification of the DSM-5 [2]. All subjects accessing our outpatient services were administered a battery of self-report questionnaires, including the Beck Depression Inventory-II (BDI-II) [23] and the Autism-Spectrum Quotient (AQ) [24].

The BDI-II is a 21-item self-report inventory measuring the severity of depressive symptoms in adults and adolescents. For each item, participants must choose which sentence (rated on a Likert scale from 0 to 3) best describes their feelings in the last two weeks. Total score reflects the severity of depressive symptomatology. The adult version of the AQ is a widely used and validated self-report instrument for assessing autistic traits in the general population. Evidence from the literature supports its good reliability and internal consistency [24]. The AQ comprises 50 items rated on a 4-point Likert scale. Higher total scores indicate greater levels of autistic traits. Subscale scores correspond to five domains: social skills, communication, imagination, attention to detail, and attention switching. Elevated scores in each domain reflect greater impairment or atypicality in the respective area.

Statistical analysis was performed using Jamovi (version 2.3.21.0) [25]. Sample characteristics were analyzed using descriptive statistics, including means and standard deviations (SDs) for continuous variables, and counts and percentages for categorical variables. Univariate analyses were conducted using non-parametric tests. A correlation matrix was used to analyze the relationships between depressive symptomatology and autistic traits, providing insights into their interdependence and associations. All tests were two-tailed, with the significance level set at 0.05.

## 3. Results

The sample comprised 64 adults diagnosed with ASD (29 females, 45.3%). The average number of years of education was 13.8 (SD = 2.24). Current cannabis use was reported by 5 participants (7.8%), and 45 individuals (77.6%) resided in urban areas. At the time of assessment, 27 participants (42.2%) were receiving pharmacological treatment, including antipsychotics, antidepressants, mood stabilizers, or benzodiazepines. General practitioners or specialists prescribed all therapies before the first evaluation.

Forty-four patients (68.7%) presented with at least one comorbid psychiatric disorder.

No significant differences were found between participants with comorbid psychiatric conditions (ASD-Comorbid) and those without (ASD-Non-Comorbid) in terms of gender, age, years of education, urbanicity, or cannabis use. Detailed descriptive and univariate statistics for the sociodemographic characteristics of the entire sample and the differences between the ASD-Comorbid and ASD-Non-Comorbid groups are presented in Table 1.

**Figure 1:**
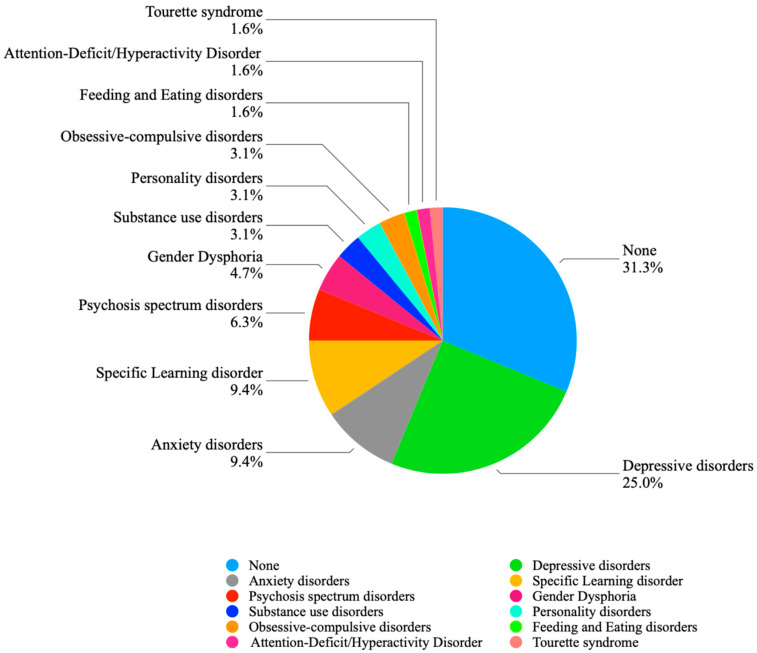
Comorbidities distribution.

Depression (*n* = 16, 25.0%) and anxiety disorders (*n* = 6, 9.4%) emerged as the most frequently reported conditions. Absolute numbers and prevalence rates of psychiatric comorbidities are detailed in Table 2 and summarized in Figure 1.

Sixty-one subjects completed the BDI-II, while 54 completed the AQ. Self-report measures are reported in Table 3. According to the BDI-II, twenty-six subjects (42.6%) reported no depressive symptoms. More than half of the participants (*n* = 35; 57.4%) reported at least mild levels of depression, with twenty-six (42.6%) showing clinically relevant moderate to severe depressive symptoms. Depressive symptoms distribution is summarized in Table 4.

Finally, a Spearman correlation analysis was conducted to explore the relationship between depressive symptoms (BDI-II total score), autism symptom severity (ADOS-2 subscales and total score), and autistic traits (AQ total score). The results showed a significant positive correlation between BDI-II and AQ scores (ρ = 0.369, *p* = 0.006), indicating that higher self-reported autistic traits were associated with increased depressive symptomatology. In contrast, no significant correlations emerged between BDI-II and ADOS-2 domains or ADOS-2 total score, although a trend-level negative association was observed with the ADOS-2 total score (ρ = −0.286, *p* = 0.060). Correlation matrix results are presented in Table 5.

## 4. Discussion

The present study aims to investigate the presence of comorbid psychiatric conditions in a sample of adult individuals with ASD referred to two outpatient services.

A high rate of psychiatric comorbidity was observed, with 44 (68.8%) participants presenting at least one additional diagnosis alongside ASD. The most prevalent comorbid conditions were depressive (25.0%) and anxiety disorders (9.4%), which together accounted for over one-third of all cases. Specific learning disorders showed a comparable prevalence (9.4%), while psychotic spectrum disorders were somewhat less frequent (6.3%). All other reported disorders occurred at a frequency below 5% each.

These findings are consistent with the existing literature. An epidemiological study conducted in a large US metropolitan region showed that the majority of adolescents with ASD (58.8%) had a co-occurring neuropsychiatric disorder [26]. A recent meta-analysis involving a sample of more than 26,000 adults with ASD reported high rates of comorbid anxiety and depressive disorders: pooled estimates indicated a current prevalence of 23% for depressive disorders and 27% for anxiety disorders, underscoring the need for routine mental health screening in this population [27]. Lai and colleagues also conducted a meta-analysis to estimate the prevalence of psychiatric comorbidities in individuals with ASD across the lifespan, reporting high rates of mood and anxiety disorders [28]. Anxiety and depressive symptoms tend to increase slightly from adolescence to middle adulthood, followed by a modest decline in older age [29]. These developmental trends underscore the importance of timely assessment and intervention.

High rates of anxiety and depressive disorders may be partly explained by the unique challenges faced by high-functioning autistic individuals, particularly in adulthood [27,28]. Individuals with Level 1 ASD and preserved cognitive functioning often face challenges in social integration, emotional regulation, and managing complex interpersonal and occupational demands. The transition to adulthood is often accompanied by social and occupational challenges, such as obtaining a driver’s license or entering the workforce. Individuals with ASD may experience greater difficulties in these domains compared to their neurotypical peers. These challenges can lead to increased stress and repeated experiences of failure or rejection, all of which may contribute significantly to the development of affective disorders, including anxiety and depression [30].

The onset of depressive disorders in individuals with ASD may also be associated with increased awareness of their core social difficulties and, in the more severe cases, this trajectory may contribute to suicidal ideation or attempts [31,32].

Increasing evidence highlights significant overlaps and high comorbidity between ASD and psychosis [12,33,34,35]. The prevalence of comorbid psychosis in our sample is consistent with recent meta-analysis [36]. ASD individuals exhibit a higher risk of developing psychosis compared to the general population [37]. However, the identification of comorbid psychosis in patients with ASD still represents a complex challenge even for expert clinicians [12]. Moreover, depressive symptoms are relatively common during the psychotic prodromal stage, which may further complicate the differential diagnosis of ASD, depression, and psychosis risk in adolescents and young adults [38].

Intercepting and preventing the onset of a psychotic breakdown in ASD might be crucial to improve treatment and prognosis of these patients. Further targeted research is needed to refine diagnostic approaches and to develop tailored interventions that address psychotic-like experiences in ASD and other neurodevelopmental disorders [39,40].

No significant differences in sociodemographic characteristics emerged between the ASD-Comorbid and ASD-Non-Comorbid groups. This finding may be primarily attributed to the limited sample size, which reduces statistical power and the ability to detect subtle group differences. Additionally, the relative homogeneity of the sample with respect to sociodemographic variables may have further limited variability. Furthermore, variables such as education level or urbanicity may not adequately capture the psychosocial complexities or environmental stressors that contribute to the development of comorbid psychiatric conditions.

A secondary objective of this study was to assess the severity of depressive symptoms in adults with ASD. Depression is characterized by core features such as depressed mood and anhedonia, alongside associated symptoms including low self-esteem, all of which contribute to functional impairment and poorer long-term outcomes in adult patients with ASD [41]. Although several studies have reported no significant gender differences in the expression of depressive symptoms among individuals with ASD [42,43], the overall evidence remains heterogeneous. Some findings from older cohorts indicate a higher prevalence of depression in females [44], while others suggest greater rates in males [45]. Consistent with the majority of studies, our results did not reveal significant differences in depressive symptomatology based on age or gender.

The accurate identification of depression in ASD is often complicated by diagnostic overshadowing, as several depressive symptoms (e.g., social withdrawal) overlap with core features of ASD [2,46,47]. Social difficulties inherent to ASD may also contribute to the development and presentation of depression, with social comparison processes playing a particularly relevant role in adolescents [48]. Moreover, concerns remain regarding the psychometric validity of standard depression measures in autistic populations. Nonetheless, Gotham and colleagues [49] demonstrated acceptable to strong internal reliability for several gold-standard tools, including BDI-II, which was used in this study.

Notably, a significant correlation was found between AQ score and BDI-II score, but no correlation between ADOS-2 scores and depressive symptoms. These findings may suggest that depression in ASD is more strongly influenced by external psychosocial stressors—such as bullying, or lack of support—rather than the severity of autistic symptoms per se. The significant correlation between BDI-II and AQ scores may indicate that the internal perception of cognitive rigidity and sensory sensitivity can contribute to emotional distress and depressive symptomatology. In contrast, the absence of a significant correlation between BDI-II and ADOS-2 scores may be partly explained by the limited variability of ADOS-2 total scores in our sample. The restricted range of ADOS-2 values, compared to the broader distribution of AQ scores, likely reduced the statistical power to detect an association, even if one exists in the population.

Our final consideration concerns the complexity of diagnosing ASD in adults. Before the expansion of diagnostic criteria in the DSM-5, many individuals were excluded from diagnosis, leading to the concept of a “lost generation” of adults with ASD [50]. Although these individuals can now seek re-evaluation, diagnosing ASD in adulthood, particularly in milder forms, remains challenging. Camouflaging and adaptive strategies further complicate the diagnostic process. Moreover, ASD rarely occurs in isolation. Comorbidities often present unique challenges in clinical practice, requiring a multidisciplinary and nuanced approach to assessment and care. Adult psychiatric services may struggle to recognize ASD, particularly in patients with comorbid psychosis, negative symptoms [12,51] and depression, highlighting the need for improved clinician training. Early detection of attenuated psychotic symptoms (APSs) in ASD [33,52] or psychopathological markers [39] during the transition from adolescence to adulthood may radically alter the course of comorbidities and improve prognosis, as APSs may require specific treatments [53]. Understanding and identifying comorbidities in ASD is critical not only for achieving accurate diagnoses, but also for tailoring effective, individualized interventions. Early identification and targeted intervention for depression represent critical opportunities to improve long-term outcomes in individuals with ASD.

## 5. Strengths and Limitations

The study has several limitations and strengths that should be considered.

One limitation of the present study is the low number of participants diagnosed with comorbid Attention-Deficit/Hyperactivity Disorder (ADHD). Although previous research has identified ADHD as one of the most prevalent comorbidities in individuals with ASD, only one participant in our sample (1.6%) received a comorbid ADHD diagnosis. This finding is likely attributable to a sampling bias. Specifically, patients referred to our adult ASD outpatient clinic typically undergo an initial evaluation in a general psychiatry setting. Individuals with suspected ADHD are often referred directly to specialized services for ADHD diagnosis and treatment, whereas those with suspected ASD are referred to our specialized clinic. Consequently, many individuals with prominent attentional or hyperactivity symptoms are filtered out before referral, leading to an underrepresentation of comorbid ADHD in our sample.

Additionally, the relatively small sample size may limit the generalizability and reproducibility of the findings in larger or more diverse populations. Finally, the cross-sectional design prevents the investigation of causal relationships or the longitudinal impact of psychiatric comorbidities and depressive symptoms on clinical course and functional outcomes in adults with ASD.

## 6. Conclusions

The present study highlights the substantial burden of psychiatric comorbidities among adults with ASD, with mood and anxiety disorders emerging as the most prevalent conditions. These findings emphasize the clinical need for comprehensive, multidisciplinary assessments that move beyond the core ASD symptomatology to include systematic screening for co-occurring psychiatric disorders. Such an approach can support the development of individualized care plans, integrating pharmacological, psychotherapeutic, and psychosocial interventions tailored to the complex needs of this population. Moreover, the recognition of psychiatric comorbidities has important implications for prognosis, quality of life, and functional outcomes, reinforcing the necessity of early detection and targeted intervention. Future research employing larger sample sizes, rigorous methodologies, and longitudinal designs is needed to address these limitations and to deepen understanding of the ASD phenotype in adults, with crucial implications for prognosis and therapeutic strategies. A more nuanced understanding of the interplay between ASD and psychiatric comorbidities holds the potential to inform personalized therapeutic strategies, reduce clinical burden, and improve long-term outcomes for adults on the spectrum.

## Figures and Tables

**Table 1:** Descriptive and univariate statistics of sociodemographic characteristics of the entire sample and divided into the “ASD-Comorbid” vs. “ASD-Non-Comorbid” groups.

Variables	Total Sample (*n* = 64; 100%)	ASD-Comorbid (*n* = 44; 68.7%)	ASD-Non-Comorbid (*n* = 20; 31.3%)	Statistics	
				Value	*p*
Age	30.9 (±8.92)	31.1 ± 8.55	30.4 ± 9.89	405 ^a^	0.612
Education	13.8 ± 2.24	13.5 ± 2.05	14.4 ± 2.56	334 ^a^	0.08
Gender					
F	29 (45.3%)	19 (29.7%)	10 (15.6%)	0.258 ^b^	0.787 ^c^
M	35 (54.7%)	25 (39.1%)	10 (15.6%)		
Cannabis Use					
Yes	5 (7.8%)	5 (7.8%)	0 (0%)	2.47 ^b^	0.314 ^c^
No	59 (92.2%)	39 (60.9%)	20 (31.3%)		
Urban Area					
Yes	51 (79.7%)	34 (53.1%)	17 (26.6%)	0.507 ^b^	0.739 ^c^
No	13 (20.3)	10 (15.6%)	3 (4.7%)		
Pharmacological therapy					
Yes	27 (42.2%)	20 (31.3%)	7 (10.9%)	0.616 ^b^	0.586 ^c^
No	37 (57.8%)	24 (37.5%)	13 (20.3%)		

^a^ Kruskal–Wallis test; ^b^ Chi-square (χ^2^) test; ^c^ The Chi-square *p*-values have been adjusted using Fisher’s exact test. Continuous variables are presented as means and standard deviations of years, and categorical variables as counts and percentages. F: female; M: male.

**Table 2:** Comorbidities distribution.

Comorbid Diagnosis	*N*	Percentage (%)
None	20	31.3%
Depressive disorders	16	25%
Anxiety disorders	6	9.4%
Specific Learning disorder	6	9.4%
Psychosis spectrum disorders	4	6.3%
Gender Dysphoria	3	4.7%
Substance use disorders	2	3.1%
Personality disorders	2	3.1%
Obsessive-compulsive disorders	2	3.1%
Feeding and Eating disorders	1	1.6%
Attention-Deficit/Hyperactivity Disorder	1	1.6%
Tourette syndrome	1	1.6%

**Table 3:** Descriptive Statistics for BDI-II and AQ Scores in the Study Sample.

Self-Reports	*N*	Missing	Mean	Median	SD	Minimum	Maximum
BDI-II Score	61	3	18.00	18	12.2	1.00	51.0
AQ Score	54	10	33.7	34	8.02	12	48

BDI-II: Beck Depression Inventory-II; AQ: Autism-Spectrum Quotient.

**Table 4:** Severity of depressive symptoms.

BDI-II Depressive Symptoms	*N*	Percentage (%)
Absent	29	42.6%
Mild	9	14.8%
Moderate	15	24.6%
Severe	11	18.0%

BDI-II: Beck Depression Inventory-II.

**Table 5:** Correlation matrix.

		ADOS-2 Communication Domain Score	ADOS-2 Social Domain Score	ADOS-2 Total Score	AQ Total Score
BDI-II Score	Spearman’s rho	−250	−0.190	−0.286	0.369
	*p* value	0.102	0.217	0.060	**0.006**

BDI-II: Beck Depression Inventory-II; ADOS-2: Autism Diagnostic Observation Schedule Second Edition–Module 4; AQ: Autism-Spectrum Quotient. Significant *p*-values are in bold.

## Data Availability

The data presented in this study are not available on request from the corresponding author due to privacy and ethical restrictions.
